# P13 Development of a consult and antimicrobial stewardship service in Manchester Foundation Trust

**DOI:** 10.1093/jacamr/dlac004.012

**Published:** 2022-02-16

**Authors:** Karen A. Devine, Wai Kein Ng, Farnaz Dave, Cathy Chow, Darcy Emery, Michael Riste

**Affiliations:** North Manchester Hospital, Manchester Foundation Trust, Manchester, UK; North Manchester Hospital, Manchester Foundation Trust, Manchester, UK; North Manchester Hospital, Manchester Foundation Trust, Manchester, UK; North Manchester Hospital, Manchester Foundation Trust, Manchester, UK; Manchester University Medical School, Manchester, UK; North Manchester Hospital, Manchester Foundation Trust, Manchester, UK

## Abstract

**Objectives:**

To prospectively audit the first year of the North Manchester infectious diseases AMS and Consult service June 2020 to June 2021 to: (i) characterize the number of patients who had antibiotic treatment narrowed, duration reduced and/or switch to oral options; (ii) assess documentation of indication and duration of antibiotic prescriptions; (iii) determine whether or not the appropriate microbiological specimens have been performed and with the lab; (iv) identify the impact on antibiotic consumption measured in defined daily dose (DDDs); and (v) establish whether base teams act on the advice given by the Consult/AMS team (data over a 6 week period).

**Methods:**

On weekdays an electronic prescribing list of all patients prescribed piperacillin/tazobactam, meropenem and ciprofloxacin was generated, and the consults team performed either a bedside or notes review. Data was collected prospectively on 730 patients on which infection was treated, which specialty managed the patient, microbiology samples sent, duration and indication of treatment documentation, and advice given by consults team. Data was then collected over a further 6 weeks looking at whether the base specialty carried out the advice of the AMS team.

**Results:**

The main indication for broad spectrum antibiotic use was urinary tract infection (151/720) and hospital acquired pneumonia (104/720). Poor documentation of antimicrobial indication and duration was observed across all specialties (277/720). Just over 50% of appropriate microbiology samples were sent (422/726). For 42% of cases (311/730), colleagues were advised to stop or reduce antibiotic duration. In 30% of cases (220/730), narrowing of antibiotic spectrum was advised. Thirty percent (219/730) were advised on IV to oral switch. There was a modest reduction in antimicrobial consumption in some specialties comparing the year prior to the introduction of the service 2019–20 to 2020–21; 17.4% reduction respiratory (7810/9137), 0.2% increase in medicine (272698/267351) and 2.1% surgery (68722/67309). In 81 patients reviewed to look at outcomes over 6 weeks, 88% of Consult/AMS team advice was actioned by the lead specialties involved in the patients’ care.

**Conclusions:**

(i) There is a substantial need for an AMS service within the hospital. (ii) Outcome data shows the ward teams engage with the service and most follow advice. (iii) The service contributed to limiting inappropriate prescribing during the COVID-19 pandemic. There was a modest rise in hospital-wide consumption. (iv) The AMS service needs to be available to provide patient equity across Manchester Foundation Trust, fostering a city-wide AMS strategy that encompasses education, guidelines, audit and quality improvement.

